# In Silico Insights into the Inhibition of ADAMTS-5 by Punicalagin and Ellagic Acid for the Treatment of Osteoarthritis

**DOI:** 10.3390/ijms26094093

**Published:** 2025-04-25

**Authors:** Austen N. Breland, Matthew K. Ross, Nicholas C. Fitzkee, Steven H. Elder

**Affiliations:** 1 Department of Agricultural & Biological Engineering, Mississippi State University, Starkville, MS 39762, USA; abreland@ufl.edu; 2 Department of Comparative Biomedical Sciences, Mississippi State University, Starkville, MS 39762, USA; mross@cvm.msstate.edu; 3 Department of Chemistry, Mississippi State University, Starkville, MS 39762, USA; nfitzkee@chemistry.msstate.edu

**Keywords:** osteoarthritis, aggrecanase, molecular docking, punicalagin, ellagic acid

## Abstract

ADAMTS-5 (aggrecanase-2) is a major metalloprotease involved in regulating the cartilage extracellular matrix. Due to its role in removing aggrecan in the progression of osteoarthritis (OA), ADAMTS-5 is often regarded as a potential therapeutic target for OA. Punicalagin (PCG), a polyphenolic ellagitannin found in pomegranate (*Punica grunatum* L.), and ellagic acid (EA), a hydrolytic metabolite of PCG, have been widely investigated as potential disease-modifying osteoarthritis drugs (DMOADs) due to their potent antioxidant and anti-inflammatory properties, but their interaction with ADAMTS-5 has yet to be determined. In this study, molecular docking simulations were used to predict enzyme–inhibitor binding interactions. The results suggest that both compounds may be able to bind within the active site via the formation of H bonds and interactions between the ligand’s aromatic rings and hydrophobic residue in the enzyme with inhibition constants of 183.3 µM and 1.13 µM for PCG and EA, respectively. Biochemical activity against recombinant human ADAMTS-5 was assessed using a dimethylmethylene blue-based assay to determine residual sulfated glycosaminoglycan (sGAG) in porcine articular cartilage. Although its loss could not be attributed to ADAMTS-5, sGAG was effectively persevered by PCG and EA. The potential conversion of PCG to EA by enzyme-catalyzed hydrolysis activity was then investigated using liquid chromatography–mass spectroscopy to determine the potential for the use of PCG and EA as a prodrug–proactive metabolite pair in the development of drug delivery systems to arthritic synovial joints.

## 1. Introduction

Globally, there were over 525 million prevalent cases of osteoarthritis (OA) in 2019, and cases had increased by 113% since 1990 [1]. During that same interval, the anatomic site-specific age-standardized prevalence rate increased for most joints, including the knee, which contributes the most to the overall burden of the disease [2]. The most prominent risk factor for OA is age, and OA is among the leading causes of disability after age 70 [3]. However, early-onset OA is on the rise, and patients younger than 55 now account for most new incident cases [3]. Other key risk factors for OA are genetics, obesity, joint injury, and female sex.

The heritability of OA has been estimated at 50% or more, and over 100 polymorphic DNA variants associated with OA have been identified [4]. Furthermore, the proteins these genes encode have wide-ranging biological functions, such as extracellular signaling, transcription factors, and cytoskeletal proteins [4]. Thus, OA is a polygenetic disease and likely involves a complex interplay of genetic factors.

Obesity is also a very important risk factor for OA, but the mechanism is not the overloading of joints gradually destroying cartilage, as once thought. Rather, obesity seems to be linked to altered innate and adaptive immune responses leading to pathological inflammation. For example, obesity affects macrophage polarization, leading to more M1 cells in comparison to M2 [5]. Although M2 is considered to be anti-inflammatory and is associated with tissue remodeling, M1 secretes pro-inflammatory cytokines such as interleukin-6 and interleukin-1β [5], both of which are drivers of OA.

Exercise is healthy for joints and generally reduces the risk of OA, but moderate-to-severe injures such as tearing of the meniscus in the knee elevates the risk of OA [3]. The meniscus transmits load across the joint and minimizes articular cartilage contact stress by distributing the load to a large surface area. It also absorbs shock and aids in joint lubrication. Post-traumatic OA is a potential consequence of the loss of these important functions.

Finally, female sex is a main risk factor for OA, particularly after the age of 50 years. Not only are women at higher risk of developing the disease, but their symptoms are often worse than those of men [3]. The reasons for the disparity are not clear and may be related to anatomical, hormonal, or behavioral differences. Because estrogen is crucial for maintaining healthy articular cartilage and subchondral bone in animal studies, it is speculated that estrogen depletion in humans could increase susceptibility to OA [6].

The symptoms of OA are pain, stiffness, swelling, and loss of range of motion in the affected joint, leading to significantly diminished quality of life and loss of mobility. In late stages, this condition often leads to total joint replacement due to the debilitating pain and near impossibility of sustaining normal daily activities. While weight loss, physical therapy, and invasive surgical options (chondroplasty, osteochondral autograft, total joint replacement, etc.) have well-documented benefits for OA patients, pharmacological interventions are not particularly effective [7,8,9]. The most prevalent intra-articular (IA) treatment options for OA (hyaluronic acid, glucocorticoids, platelet-rich plasma) are slightly beneficial for pain and function in knee OA [10]. However, relief from symptoms is typically temporary, with no long-term effects on disease progression [11]. Therefore, research into novel treatment options is needed to help reduce the financial and humanistic impacts of this disease.

Our laboratory has previously explored punicalagin (PCG), an active antioxidant polyphenol found in pomegranates, as a potential disease-modifying osteoarthritis drugs (DMOADs) [12,13]. It is attractive for its polypharmacological properties, as it can act on multiple targets pertaining to multiple pathophysiological mechanisms involved in OA. PCG has been shown to inhibit the production of pro-inflammatory cytokines, including TNF-α, IL-6, and IL-1β, by attenuating NF-κB/iNOS/COX-2/TNF-α/FOXO3 and mitogen-activated protein kinase (MAPK) signaling pathways [14,15,16,17,18,19]. The suppression of NF-κB/iNOS/COX-2/TNF-α also lowers the production of PGE2, which when left unchecked enhances the expression of catabolic enzymes and accelerates extracellular matrix (ECM) degradation [20]. Another chondroprotective action of PCG is the scavenging of reactive oxygen species. It possesses multiple phenol groups that can participate in redox reactions by donating hydrogen atoms to oxidizing agents. Thus, it has potent electron-donating capacity, enabling it to efficiently reduce and detoxify free radicals. PCG could also be chondroprotective through binding to cartilage collagen and limiting access to degradative enzymes. PCG binds to collagen type II with high affinity, which occurs through the formation of multiple hydrogen bonds (PCG has 17 hydroxyl groups) in addition to π-π and electrostatic interactions [21]. Finally, PCG can directly inhibit the secreted enzymes that degrade the cartilage ECM in OA. It was previously established that PCG forms complexes with collagenases, including MMP-13, in solution and inhibits their enzymatic activity [12,21].

There is growing evidence that PCG is effective for the treatment of OA. Orally administered pomegranate fruit extract has been shown to lessen the severity of induced OA in rats and rabbits [16,22]. Daily intraperitoneal doses of PCG at 50 mg/kg significantly reduced paw edema in an adjuvant-induced arthritis rat model [21]. As part of an investigation of PCG as a potential therapeutic compound for relieving rheumatoid arthritis, PCG was found to reduce the TNF-α-induced expression of IL-1β, IL-6, and MMP-13 in fibroblast synoviocytes [23]. Such studies point to PCG as a promising DMOAD. Moreover, PCG safety is well established. For example, PCG cytotoxicity was determined for the Vero, Hep-2, and A-549 cell lines, and all CTC_50_’s were over 450 µM [24]. Furthermore, hematological and histopathological analyses of rats fed a 6% PCG-containing diet for 37 days indicated no systemic toxicity [25]. Likewise, the histology of major organs revealed no evidence of systemic toxicity in our previous study [12].

The purpose of this study is to investigate the potential for PCG to inhibit ADAMTS-5, as might occur through binding to its active site. In OA, cartilage proteoglycan is subject to attack by a subgroup of proteases known as ADAMTS’s (a disintegrin-like and metalloprotease domain with thrombospondin type 1 repeats). Of the 19 members within this subgroup, ADAMTS-4 (aggrecanase-1) and ADAMTS-5 (aggrecanase-2) are known to be the primary contributors to the removal of aggrecan in OA [26]. Specifically, they cleave the peptide between Glu-373 and Ala-374 [27]. The high density of negatively charged anionic groups found within aggrecan generates a significant osmotic swelling pressure that resists compressive loading [28]. Thus, the preservation of proteoglycan is essential for overall joint health. Due to aggrecan’s vital role in the maintenance of homeostatic cartilage function and the known role of ADAMTS proteases in aggrecan removal, the development of a potent, selective, and non-cytotoxic inhibitor for ADAMTS-4 and ADAMTS-5 represents a potential method of slowing the progression of OA. Additionally, experiments that induce gene-targeted deletion of the catalytic domains of ADAMTS-4 and ADAMTS-5 in mice have suggested that the inhibition of ADAMTS-5 protects against OA progression [29].

**Figure 1:**
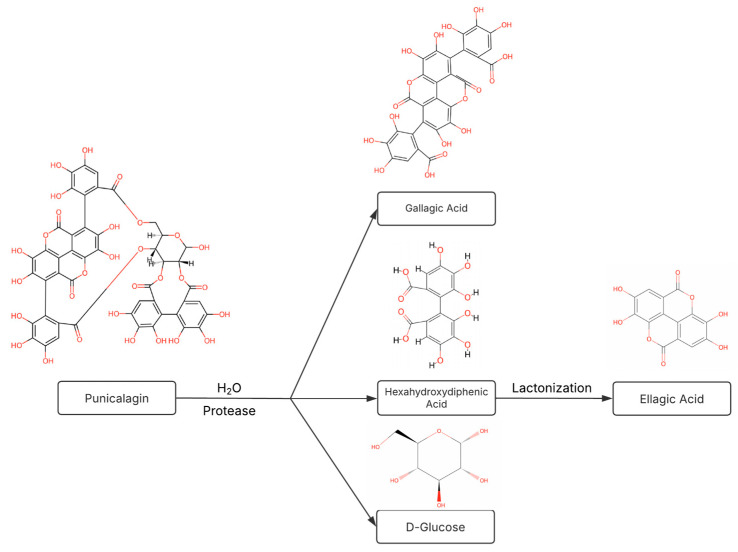
Punicalagin (PCG) degradation pathway. Hydrolysis of PCG’s ester bonds via acid/base catalyzed hydrolysis or active cleavage by in vivo proteases may produce metabolites with additional unique therapeutic effects.

A study of PCG’s ability to inhibit ADAMTS-5 would not be complete without consideration of ellagic acid (EA), PCG’s primary metabolite. The oral bioavailability of PCG is low due to hydrolysis in the stomach and intestine, which yields EA [30,31]. However, significant effort has been invested in developing long-term intra-articular and oral delivery systems for PCG [13,32]. The production of EA results from the release of hydrolytic enzymes by gut bacteria, such as *Lactobacillus plantarum.* These enzymes, known as tannases, are responsible for cleaving ester bonds found in PCG to yield hexahydroxydiphenic (HHDP) acid, d-glucose, and gallagic acid (GA). However, HHDP acid is an unstable structure that is spontaneously converted to EA via lactonization, as seen in Figure 1 [33]. HHDP acid, d-glucose, and GA are also likely metabolic derivatives of PCG outside of the digestive system due to the susceptibility of ester linkage hydrolysis in biological environments, as well as the prevalence of ester bond cleavage via protease activity [34,35]. Even when PCG was released from polycaprolactone implants placed subcutaneously in rats, only EA was detected in the plasma (no intact PCG) [36]. EA, a polyphenol that occurs naturally in berries and nuts, has also been widely researched as a potential DMOAD [37]. It has been previously demonstrated to inhibit IL-1β-induced TNF-α, IL-6, and inducible nitric oxide synthase expression [38].

The goals of this study can be divided into three key objectives that were investigated consecutively, namely predicting drug interactions of the PCG metabolome with ADAMTS-5, analyzing the chondroprotective properties of the PCG metabolome against ADAMTS-5 in vitro, and assessing the PCG-to-EA conversion rate in experimental conditions. By identifying the interactions between these compounds and ADAMTS-5, a key enzyme involved in OA progression, this work supports the characterization of PCG and EA as prospective DMOADs.

## 2. Results

In the investigation of potential binding pockets within ADAMTS-5 using DoGSite within ProteinsPlus, a druggability cutoff of 0.5 was utilized, which resulted in three potential binding pockets detailed in Table 1 [39]. A further characterization of the biochemical properties of all potential ligands assessed in this study is summarized in Table 2. Log P, determined as the octanol–water affinity by XLogP3, represents the lipophilicity of a particular molecule and contributes to the potential for each ligand to form hydrophobic interactions with enzyme residue, as well as its membrane permeability. The polar surface area (PSA) influences the potential for each ligand to form H bonds.

### 2.1. Molecular Docking Simulations

**Figure 2:**
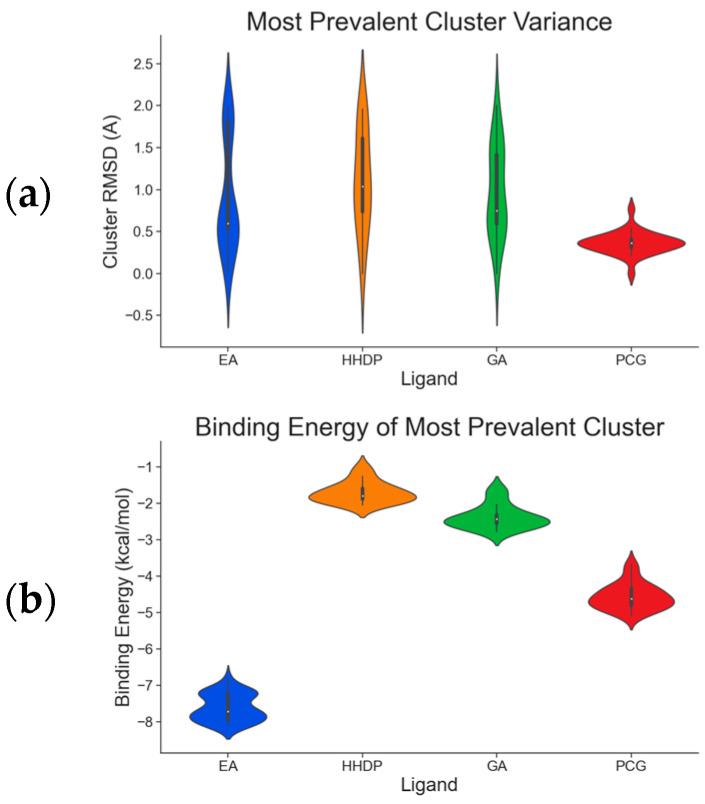

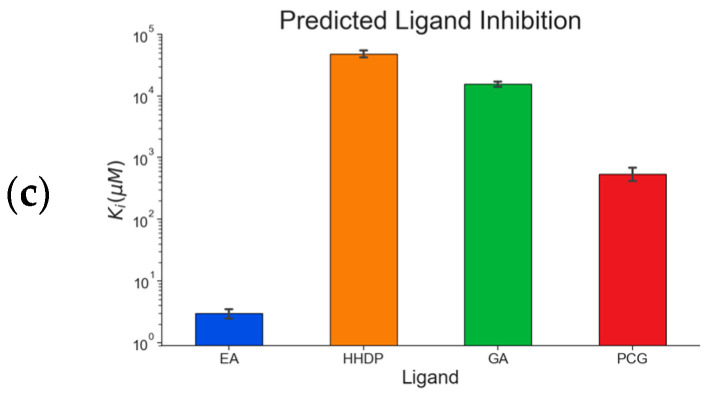
Characterization of the most prevalent ligand clusters. (**a**) RMSD (Å) of enzyme–ligand conformation clusters. (**b**) Free energy change in binding. (**c**) Predicted ligand inhibition constant (K_i_).

According to our simulations, gallagic acid (K_i_ = 9.16 mM) and hexahydroxydiphenic acid (K_i_ = 31.81 mM) were predicted to have a very low potency for inhibiting ADAMTS-5 (Figure 2). For these two ligands, the most populated cluster did not coincide with the lowest binding energy cluster. In GA’s optimal binding conformation, we observed that a few H bonds were predicted to form between phenol groups and the carboxyl groups of Leu-443 (2.71 and 2.68 Å) and Ala-448 residue (2.59 Å) (Table 3). Additionally, the estimated binding free energy of GA (−2.78 kcal/mol) included a significantly positive torsional free energy contribution (+4.77 kcal/mol).

For HHDP, H bonds were formed between ligand phenol groups and His-410 (2.83 Å) and His-420 (3.17 Å) imidazole side chains along with one formed between a carboxyl oxygen and Leu-379 (2.75 Å) amino group. The length of these bonds and the prediction of high torsional free energy (+3.28 kcal/mol) relative to the total predicted free energy (−2.04 kcal/mol) suggests an unstable conformation.

Ellagic acid and punicalagin portrayed a significantly more favorable predicted binding affinity for ADAMTS-5, with their optimal and most prevalent binding cluster coinciding. For EA’s optimal conformation (Ki = 1.13 µM), H bonds were observed between hydroxyl groups O4 and O5 and the carboxyl group of Glu-411 (2.59 and 2.51 Å, respectively), as well as between O5 and the aldehyde group of Gly-380 (2.50 Å). The bond between Glu-411 and O5 was by far the most common bond formed in the analysis, occurring in 40 of 50 conformations for EA. A fourth H bond was also formed between an ester oxygen, O6, and the amino N-H of Leu-443 (2.62 Å). A significantly lower binding energy (−8.11 kcal/mol) and torsional free energy (+1.19 kcal/mol) were also calculated. PCG’s optimal binding conformation (Ki = 183.3 µm) predicted the formation of H bonds between phenolic O16, O19, O21, and O30 and the carboxylic oxygen of Gly-401 (2.49 and 2.95 Å, respectively, His-403 (3.0 Å), and Leu-443 (3.30 Å). Additionally, this conformation resulted in a binding energy of −5.10 kcal/mol and a torsional free energy of +5.07 kcal/mol.

**Figure 3:**
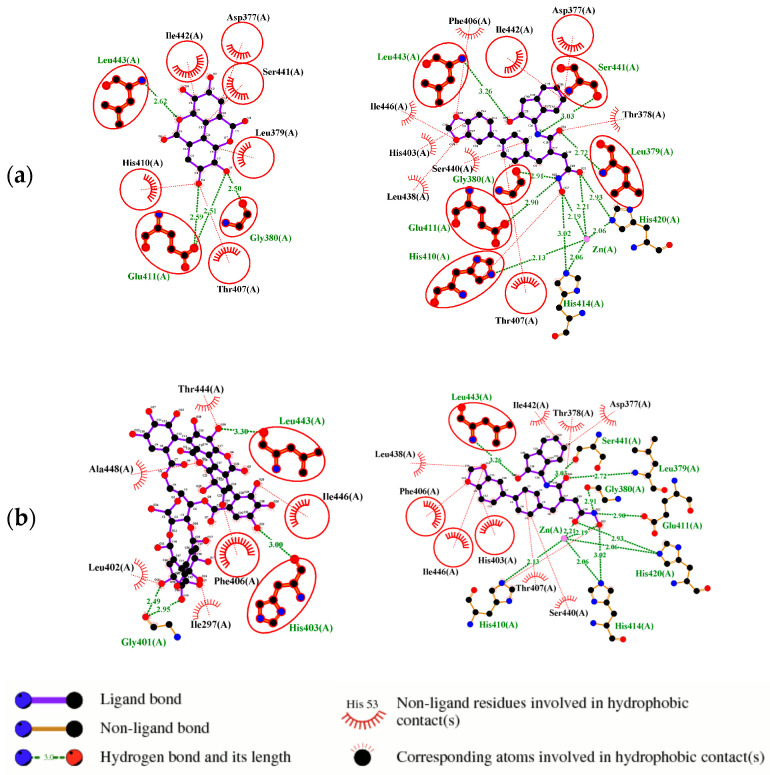
Ligand binding comparison between PCG, EA, and a known ADAMTS-5 inhibitor, with all common interacting residues highlighted in red. (**a**) Comparison of the binding interactions between EA and A2N reveals a significant degree of overlap, with all interactions of EA also being present in A2N’s binding interactions. (**b**) PCG and A2N exhibited significantly fewer common interactions.

**Figure 4:**
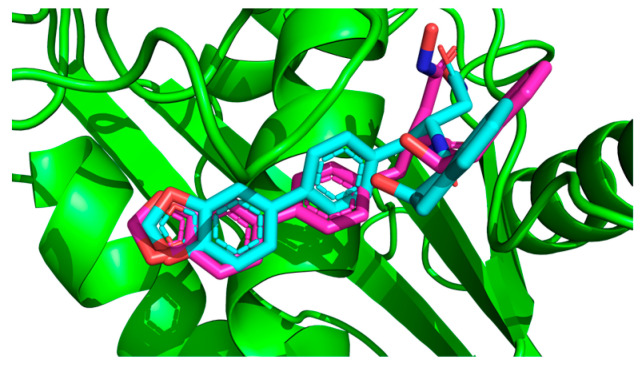
Superimposition of redocked GLPG1972/S201086 (pink) onto a co-crystallized complex (teal) in the active site using PyMOL [41] (RMSD = 1.961 Å).

The conformations for EA and PCG were also compared to the interactions of GLPG1972/S201086 (A2N), a known potent competitive inhibitor of ADAMTS-5 [40]. This showed that there is a significant overlap between the interactions of EA and A2N but only a small overlap between PCG and A2N (Figure 3). Very similar hydrophobic interactions occurred between the aromatic rings present in both EA and A2N, while similar H bonds were formed between polar phenol groups on EA, as seen in A2N’s interaction diagram. Redocking of the reference ligand, A2N, resulted in an RMSD of 1.961 Å when the conformations were superimposed (Figure 4).

### 2.2. ADAMTS-5 Inhibition

**Figure 5:**
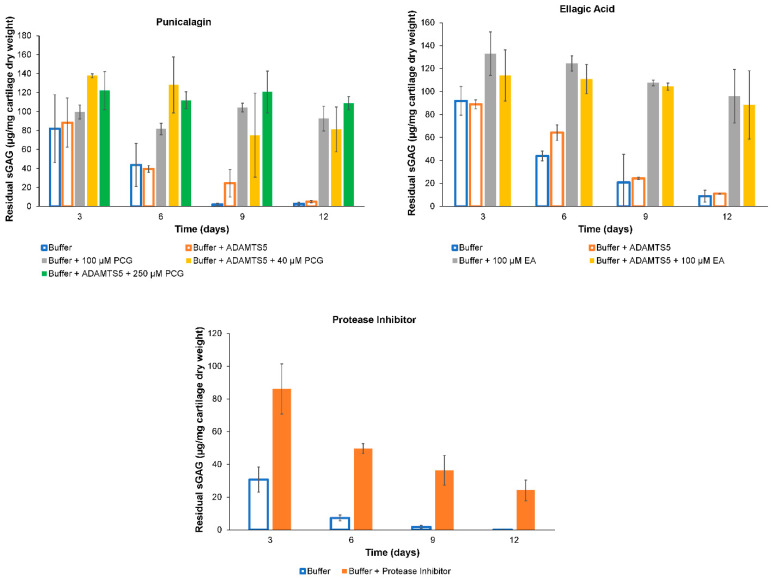
Residual sGAG in porcine articular cartilage disks after exposure to 0.4 µg/mL ADAMTS-5. Results for each group (n = 3) are displayed as mean ± standard deviation. Within each chart, groups represented by open bars are a statistically homogeneous group, as are groups represented by solid bars. Thus, groups represented by open bars are significantly different from those represented by solid bars (two-way ANOVA, *p* < 0.001).

Following the results of the molecular docking study of ADAMTS-5 with PCG and its established metabolites, the compounds with the highest predicted inhibition potential were examined for their ability to inhibit the ADAMTS-5-mediated removal of sGAG from porcine articular cartilage disks in vitro. Due to the focus on the therapeutic effects of PCG, it was tested at two different concentrations of 40 µM and 250 µM. As shown in Figure 5, disks that were incubated with PCG or EA retained substantially more sGAG than the disks incubated without them. In the case of PCG, the inhibitory effect was not dose-dependent. Disks treated with 0.4 µg/mL rhADAMTS-5 did not lose significantly more sGAG than those incubated in buffer alone. General protease inhibition was found to significantly increase the amount of residual sGAG over buffer alone. Although the predicted inhibitory potency of PCG and EA differed greatly, their demonstrated abilities to prevent aggrecan removal were not significantly different (*p* = 0.574).

### 2.3. Histology

**Figure 6:**
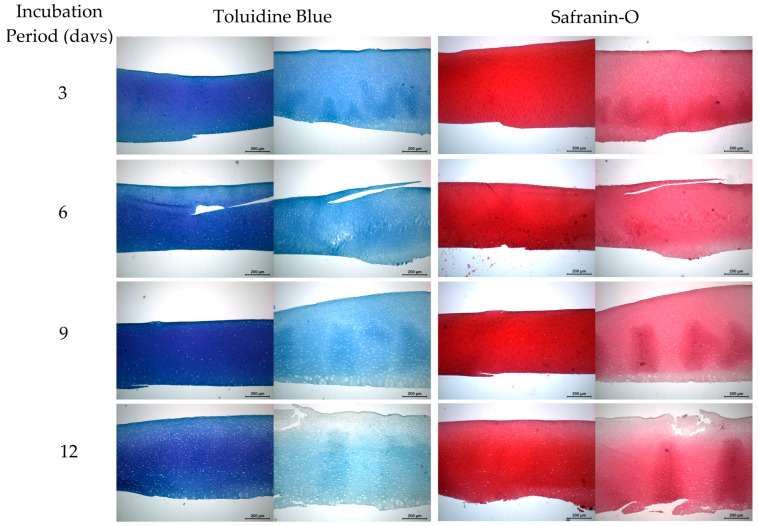
Effects of 250 µM punicalagin (PCG) treatment on glycosaminoglycan (GAG) retention following incubation with ADAMTS-5 (0.4 µg/mL). For each incubation period and staining technique, left images represent treated groups while right images represent a non-treated group. Scale bars = 200 µm.

The chondroprotective properties of PCG were further analyzed using toluidine blue and safranin-O staining to examine sGAG retention following incubation with ADAMTS-5. This resulted in a visually significant difference in staining saturation between the PCG treatment and non-treatment groups, as seen in Figure 6.

### 2.4. Hydrolyzability of Punicalagin

**Figure 7:**
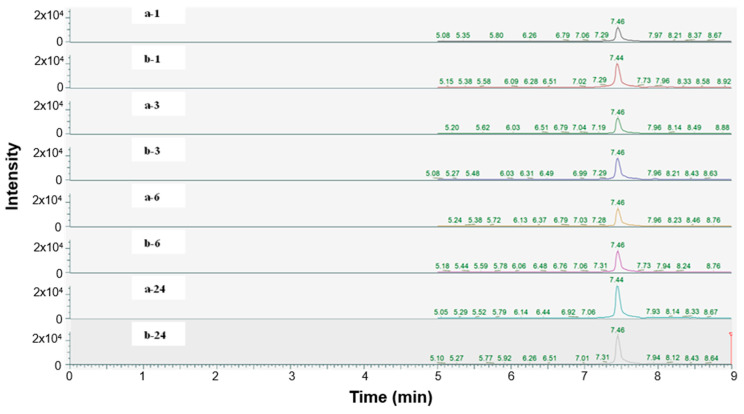
LC-MS/MS chromatograms of EA in buffer solution (**a**) and enzymatic buffer (**b**) incubated at 1, 3, 6, and 24 h intervals.

The relative concentration of EA in solution was analyzed utilizing mass spectrometry coupled to UPLC to determine if ADAMTS-5 catalyzed the hydrolysis of PCG. EA concentration was quantified by measuring the intensity of the SRM transition *m*/*z* 300.9 > *m*/*z* 283.8 at a retention time of 7.44–7.46 min. The concentration of EA did not vary significantly between enzyme- and buffer-incubated groups over time (Figure 7). Additionally, the rate of PCG hydrolysis in solution with ADAMTS-5 decreased as incubation duration increased. This is signified by the b/a ratio, which represents the rate of hydrolysis of the enzymatic group to the control group and is reported in Table 4.

## 3. Discussion

While the initial cause of OA remains unclear, its progression is principally a result of the body’s immune response to localized and systemic pro-inflammatory signaling. Therefore, inflammatory cytokines and immune cells play a key role in mediating OA and can help guide the development of treatments that mitigate the disease’s effects. The recruitment of leukocytes (macrophages, neutrophils, T-lymphocytes, and B-lymphocytes) that secrete cytokines like IL-1β, TNFα, and IL-6, which are known to drive local inflammatory cascades, is a key marker in the early stages of OA [42]. These pro-inflammatory cytokines are known to stimulate the production of matrix metalloproteinases (MMPs) and ADAMTSs that are responsible for the degradation of the ECM [43]. The prevention of this degradation by way of direct enzymatic inhibition represents a potential method of augmenting disease progression.

Aggrecanase inhibitors are generally classified as either zinc chelators, which bind directly to the active site and chelate the catalytic Zn^2+^ ion, or exosite inhibitors, which bind externally to the active site [44]. Zinc-chelating inhibitors are commonly far more potent but may run into issues of enzymatic selectivity due to conservation between members of the ADAMTS enzymatic family. Among the potential binding locations identified in this study, pocket P1, which is partially formed by the highly lipophilic S_1_’ subsite, exhibited notable hydrophobicity. Since high hydrophobicity is a common characteristic of druggable binding sites; this suggests the potential for strong hydrophobic interactions with docked ligands [45]. P1, representing the enzymatic active site, was found to have the most optimal binding potential. This was primarily determined from the pocket volume, surface area, hydrophobicity, and potential to form H bonds. While many ADAMTS-5 inhibitors are known to bind within the active site, neither P2 nor P3 coincided with known exosite inhibitor binding locations.

Zinc-chelating inhibitors, which constitute the vast majority of ADAMTS-5 inhibitors in the literature, commonly consist of an aromatic backbone, which can interact with the lipophilic S_1_’ subsite and a zinc binding group (ZBG). Hydroxamic acid, carboxylic acid, and pyrimidinetriones are some of the most utilized ZBGs due to their polar affinity for binding to Zn^2+^ ions [46]. The importance of ZBG in inhibition can be seen in A2N (IC_50_ = 19 nM), an ADAMTS-5 inhibitor that has been evaluated in phase II clinical trials [40]. Carboxylate-based MMP and ADAMTS inhibitors usually have lower potency and potentially limited in vivo efficacy. The presence of carboxylate groups in EA demonstrates their potential as ZBGs and justifies investigating the P1 pocket as the most likely inhibitory binding location. Phenolic oxygens present in PCG and EA are not widely researched for use as ZBGs in ADAMTSs, but they have been investigated in the inhibition of histone deacetylase [47]. This fact motivates future work examining PCG and EA’s ability to function as a zinc-chelating inhibitor of ADAMTS-5.

The moderately high predicted Log P values for EA and GA (Table 2) support the expectation of hydrophobic interactions within the active site of ADAMTS-5 and prompted the selection of P1 as the docking location for our molecular docking study. This also suggests potential for enhanced cell permeability [48]. Additionally, the significantly smaller volumes of P2 and P3 may limit the binding ability, especially for large biomolecules like PCG. Others may be exploring an effective exosite inhibitor of ADAMTS-5, but to our knowledge, no effective inhibitors have been identified with binding locations examined in this study. Exosite inhibitors may be more selective due to the highly conserved S_1_ and S_1_’ domains among ADAMTS proteases [49]. A lack of selectivity could lead to broad-spectrum inhibition of multiple MMPs and ADAMTSs and off-target toxicity [44].

Our molecular docking results have provided significant insight into the potential for punicalagin and its metabolites to play a role in inhibiting the progressive degradation of the cartilage ECM by ADAMTS-5. However, it should be noted that molecular docking, while proficient in predicting ligand–protein interactions, faces many challenges that limit the application of its results. In particular, docking may fail to accurately predict the coordination geometry and dynamics of metal chelation. In the context of catalytic zinc ions crucial for ADAMTS-5 function, this analytical method may have been insufficient for predicting the binding of HHDP and GA to the Zn^2+^ ion coordinated by Glu-411, His-410, His-414, and His-420. However, further work could be performed to implement customized coordinate parameters for the ions crucial in ADAMTS-5’s enzymatic function to investigate metal chelation in the context of enzyme inhibition. Additionally, the use of rigid body docking in this study may lead to excessively conservative consideration of steric clashes and cannot accommodate subtle changes that can happen in a protein pocket during the binding of a ligand [50].

The resulting protein–ligand structures discovered in this study can only serve as a guided approximation of the potential interactions between the two molecules. Precise determination of the bound conformation requires a different methodology. For example, refinement of a crystallographic protein structure or NMR-derived constraints may be used to identify ligand interactions. Additionally, alternate docking methods, such as ensemble docking or consensus docking, may be utilized to improve predictive strength. However, these methods fall outside of the scope of this studies’ aims.

ADAMTS-5 inhibition experiments and histological imaging were performed to further assess the predicted interaction from our molecular docking studies. However, it was found that disks treated with 0.4 µg/mL rhADAMTS-5 did not lose significantly more sGAG than those incubated in buffer alone. It was hypothesized that any effect of ADAMTS-5 was overwhelmed by catabolic enzymes released from chondrocyte lysosomes upon cell lysis. Protease inhibition significantly increased the amount of residual sGAG, supporting the hypothesis.

Additionally, PCG lacked a dose-dependent inhibition of sGAG degradation. The lack of dose dependence could have resulted from enzyme saturation, where a plateau in inhibitory effect is reached below the minimum dose tested. In a previous study, PCG was found to inhibit the IL-1β-mediated release of GAG from cartilage explants in a dose-dependent manner within the range of 0.1–10 µM [21]. Future studies will determine whether the PCG inhibition of sGAG-degrading enzymes is dose-dependent by testing concentrations in the range of 0.1 to 250 µM.

Despite our inability to confirm that PCG prevented ADAMTS-5-mediated GAG loss, a histological examination of sample disks utilizing both safranin-O and toluidine blue staining provided further confirmation of the chondroprotective properties of PCG through visualization of differential staining intensity. Both staining modalities demonstrated increased GAG retention, as evidenced by more intense and uniform staining. The difference in staining quality also exhibited a time-dependent behavior, providing further evidence of protection PCG conveyed during long-term incubation periods. In future work, we aim to develop a new assay for ADAMTS-5 activity that will facilitate high-throughput screening of potential inhibitors.

Inhibition of sGAG degradation adds to the polypharmacologic effects of PCG. These include the various anti-inflammatory benefits PCG has been shown to have, such as reducing the expression of inflammatory cytokines by fibroblast synoviocytes and reduced collagen degradation via MMP-13 via the formation of inhibitory enzyme complexes [12,15,23]. Further investigation of the potential impact PCG has on other members of the ADAMTS enzymatic family is another important topic of investigation. Due to the conservation of the catalytic structure between these enzymes, multi-targeted enzyme binding should be considered.

While a direct safety analysis is not conducted in this study, significant work has been performed to establish the safety of PCG as a pharmacological intervention. ADME profiling of PCG revealed no concerns about distribution (due primarily to its water solubility), metabolism, excretion, or toxicity [51]. Additionally, a hematological and histopathological analysis of rats fed a 6% PCG-containing diet for 37 days indicated no systemic toxicity [20,21]. Likewise, histology of major organs yielded no evidence of systemic toxicity in a previous study in our lab [12].

## 4. Materials and Methods

### 4.1. Materials

PCG (98%) was from Chengdu Biopurify Phytochemicals Ltd. (Chengdu, China). EA (95%) was obtained from Caymen Chemical (Ann Arbor, MI, USA). Recombinant human ADAMTS-5 was from R&D Systems (Minneapolis, MN, USA). Dsulfoxide (DMSO), Tween-20 surfactant, and CHAPS detergent were from Sigma-Aldrich (St. Louis, MO, USA). The Blyscan™ Glycosaminoglycan Assay kit was obtained from Biocolor (Carrickfergus, UK). LC-MS solvents (water, acetonitrile) and LC-MS grade formic acid were from Fisher Scientific (Waltham, MA, USA).

### 4.2. Ligand Characterization

Molinspiration, a web-based cheminformatics server, was utilized to determine the molecular volume and total polar surface area (PSA). Additionally, XLogP3 version 3.2.2 was used to predict the Log P of punicalagin, gallagic acid, hexahydroxydiphenic acid, and ellagic acid. These were then compared to a known inhibitor of ADAMTS-5, A2N [40]. The partitioning coefficient, P, was calculated as the octanol–water affinity ratio for each ligand.

### 4.3. Enzyme Pocket Characterization

To predict binding locations for punicalalgin, gallagic acid, hexahydroxydiphenic acid, and ellagic acid, the surface biochemical properties of ADAMTS-5 were analyzed. For the enzymatic analysis, a crystallographic structure of ADAMTS-5 (PDB ID: 3LJT [52]) was uploaded to the University of Hamburg’s ProteinsPlus server [39]. The DoGSite tool, which utilizes the Difference of Gaussian filter to detect potential binding pockets, was used to collect data on the volume, surface area, hydrophobicity, H bond formation sites, and amino acid polarity ratios of enzymatic binding pockets [53].

### 4.4. Molecular Docking Simulations

According to the convention developed by Schechter and Berger, protease active sites are divided into “subsites” labeled S_n_, S_n+1_, S_n+2_, etc., from the cleaved bond to the N-terminal residue and S’_n_, S’_n+1_, S’_n+2_, etc., from the cleaved bond to the C-terminal site [54]. Similarly, substrate regions are labeled P_n_ and P’_n_ in each direction. From the analysis of crystallographic structures of ADAMTS-5 in the Protein Data Bank (PDB), the structures of the S_1_ and S’_1_ domains are integral for the binding of competitive inhibitors [40]. These domains form a characteristic fold that facilitates the coordination of a zinc ion that is essential for aggrecanase activity. The pocket of the active site in ADAMTS-5 is characterized by three histidine residues (His-410, His-414, and His-420) and a glutamic acid residue (Glu-411) that are involved in binding to a zinc ion that facilitates the cleavage of the aggrecan substrate. Figure 7 depicts the location of these residues within the overall tertiary structure of ADAMTS-5 and their relative location to the bound zinc ion. In particular, the lipophilic S_1_′ pocket (amino acids 437–451) can assume different conformations based on the bound inhibitor [44].

AutoDock Suite version 4.2.6 was used to predict the binding potential and binding conformations of PCG, GA, HHDP, and EA within the active site of ADAMTS-5 through an analysis of entropy loss upon binding. The 3D structures for these ligands were each downloaded from PubChem in .sdf format, the only exception being PCG. Due to its large molecular weight, the structure for PCG was downloaded from MolView in .mol format. The structure of ADAMTS-5 was obtained from the PDB ID 3LJT [52].

First, the PDB structure of ADAMTS-5 was imported, and all water molecules were then removed from the structure. All non-enzymatic atoms were then removed from the structure. Hydrogen atoms were generated using AutoDock software. During all docking experiments, enzymatic residues were treated as rigid. Ligand flexibility was accounted for by allowing all available torsions to rotate freely. Rotatable bonds were attributed and configured with ADT version 1.5.7, which resulted in 11 torsions for HHDP, 4 for EA, 16 for GA, and 17 for PCG.

**Figure 8:**
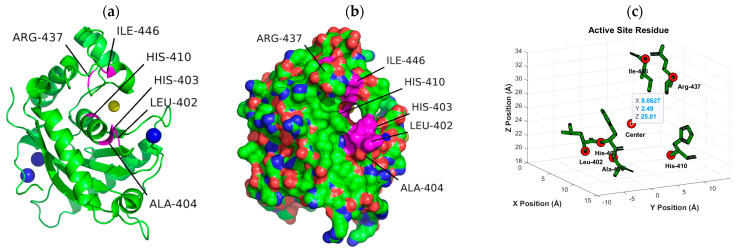
Location of residue (in pink) used to define docking region in ADAMTS-5 3D structure (Ca^2+^: blue, Zn^+^: yellow). Location of residue highlighted in pink on ribbon (**a**) and surface (**b**) depiction of enzyme. (**c**) Cartesian position of each residue with location of first atom of each residue and overall center highlighted with red circles.

The interaction calculations were made within a rectangular prism using the parameters in Table 5. The 3D coordinates used to define the center of the grid box were set to (8.063, 2.49, 25.01) (Å) and determined from the average of the 3D coordinates of the first atom from 6 residues within the S_1_ and S’_1_ domains of ADAMTS-5 (Leu-402, His-403, Ala-404, His-410, Arg-437, Ile-446). These residues were selected due to their role in the binding of established competitive inhibitors of ADAMTS-5 and the formation of the enzymatic active site [40]. This decision was also supported by the predictive analysis performed using ProteinsPlus [39]. The relative locations of these residues can be seen in Figure 8, with the cartesian center of each residue also depicted. Minimum Van der Waals and hydrogen bonding energies were computed using AutoDock parameters, and electrostatic interactions were evaluated with a distance-dependent dielectric function [55]. Docking solutions were determined using the AutoDock Lamarckian Genetic algorithm with default parameters, except for the number of genetic algorithm runs and the population size of each evaluation. For this experiment, these parameters were set to 50 and 300, respectively. The 50 resulting docking conformations were clustered with a root mean square difference (RMSD) tolerance of 2.0 Å. Optimal clustering and binding conformations were analyzed in greater detail and visualized with the use of LigPlot+ version 2.2.8 [56] and PyMol version 2.6 (Schrodinger, LLC, New York, NY, USA) [41]. Docking parameters were validated through redocking of the reference ligand, A2N (PDB ID: 3LJT [52]), utilizing the same parameters and determining the superimposed RMSD between the conformations using DockRMSD (version 0.4.2).

### 4.5. ADAMTS-5 Inhibition

Based on the results of the molecular docking study, PCG and EA were determined to be the most likely inhibitors of ADAMTS-5. They were tested in an in vitro system involving the ADAMS-5-mediated removal of GAG from porcine articular cartilage. Fresh porcine femurs were obtained from a local meat processor. Full-thickness articular cartilage disks of Ø5 mm articular cartilage were harvested from the distal femur using a biopsy punch and scalpel. They were then washed for 30 min in PBS in a Thermo Scientific MaxQ 4000 Incubated/Refrigerated Shaker (Marietta, OH, USA) at 100 RPM and 37 °C for 30 min followed by an additional 10 min wash with distilled H_2_O. After lyophilization, the disks were individually weighed and placed in 1.5 mL microcentrifuge tubes.

Human recombinant ADAMTS-5 was reconstituted at a concentration of 0.4 µg/mL in a 50 mM Tris, 100 mM NaCl, 5 mM CaCl_2_, 0.05% Tween-20 (PCG assay) or 50 mM Tris, 100 mM NaCl, 5 mM CaCl_2_, 0.05% CHAPS (EA assay) buffer solution at a pH of 7.5. The cartilage disks were incubated in 0.12 mL/mg of this buffer. This volume was determined from the average mass of all disks used in each assay. For inhibition experiments, EA was added to a final concentration of 100 µM, while PCG was analyzed at final concentrations of 40 and 250 µM. All the microcentrifuge tubes were kept in an orbital shaker at 100 RPM and 37 °C. Triplicate samples were collected at 3-day intervals and kept at −20 °C to prevent further degradation. The buffer solution was then replaced in all remaining samples. Triplicate samples incubated in the enzyme without PCG or EA were also collected at each time interval. After 12 days, all disks were freeze-dried and re-weighed. They were then digested overnight in 1 mL of a solution containing 0.2 mM papain and 13 mM L-cysteine at 60 °C. The papain solution was then diluted with PBE, and a 1,9-dimethyl methylene blue assay (Blyscan glycosaminoglycan assay kit) was used to quantify the amount of GAG remaining in the disks after enzyme treatment. To confirm that the enzyme was responsible for the loss of GAG, a similar assay was performed on additional disks that had been incubated for 12 days in the same buffer as ADAMTS-5, with replacement every third day. They were compared to untreated control disks (n = 3 per group). Data were analyzed by a two-factor ANOVA (α = 0.05), the factors being treatment group and time. When the factor had more than two levels, multiple pairwise comparisons among levels were made using the Tukey post hoc test, with *p* < 0.05 indicating a statistically significant difference.

### 4.6. Histology

All histology procedures were carried out on sections from two different cartilage disks in each group. Following previously detailed incubation periods in reconstituted ADAMTS-5 buffered solution, untreated and 250 µM PCG cartilage disks were fixed in 10% neutral buffered formalin. Paraffin-embedded sections were stained with safranin-O and toluidine blue to demonstrate GAG in the ECM and counterstained with hematoxylin to show cell nuclei. Images were captured on a Leica DM2500 microscope equipped with a DFC420 C camera (Leica Microsystems, Deerfield, IL, USA).

### 4.7. Hydrolyzability of Punicalagin

Human recombinant ADAMTS-5 was reconstituted in a 50 mM Tris, 100 mM NaCl, 5 mM CaCl_2_, and 0.05% CHAPS buffer solution at a concentration of 0.4 µg/mL (pH 7.5). CHAPS was utilized as a surfactant for the reconstitution buffer over Tween-20 due to its compatibility with LC-MS (liquid chromatography–mass spectrometry) analysis. The same buffer without ADAMTS-5 was used for all control groups. Separate 0.5 mL volumes of the ADAMTS-5 buffer and control buffer were added to 1.5 mL microcentrifuge tubes, as well as 0.1 mM of PCG. Tubes were maintained in an orbital shaker at 100 RPM and 37 °C. In total, 0.1 mL samples of the buffer with PCG were taken at 1, 3, 6, and 24 h and stored in separate 1.5 mL centrifuge tubes at −20 °C until use. This was repeated with EA by collecting a sample at 0 and 24 h to examine the hydrolysis of PCG to EA in an aqueous solution with ADAMTS-5. Equal volumes of methanol were added to all samples to prevent further enzymatic degradation of EA and PCG in solution and the samples stored at −20 °C prior to analysis. Aliquots (5 µL) of the quenched reactions were injected onto an LC-MS/MS (Thermo Quantis triple quadrupole interfaced with a Waters Acquity UPLC system). The samples were chromatographed on a BEH C18 column (100 mm × 2 mm, with a guard) (Waters Corporation, Milford, MA, USA) using the mobile phase and gradient system described in Elder et al.’s study (2024) [13]. EA was detected using the selected reaction monitoring (SRM) transition *m*/*z* 300.9 > *m*/*z* 283 and quantified by integrating the area under its chromatographic peak.

## 5. Conclusions

Following a molecular docking study examining binding affinity with ADAMTS-5, EA and PCG were identified as novel moderately potent inhibitors. This study also elucidated the specific chemical properties of each ligand that promote and prevent higher binding potential within the enzymatic active site. Subsequent inhibition assays confirmed that both PCG and EA significantly reduced the removal of sGAG from articular cartilage. The effect seems likely to have been the result of protease inhibition, but the experiments could not confirm that ADAMTS-5 was among the proteases inhibited. Experiments also did not reveal whether inhibition occurs through direct enzymatic binding or via an indirect method of protection through binding with sGAG within cartilage. Therefore, further investigation is necessary to validate the predicted binding mode proposed in this study and to explore additional mechanisms of action. Additionally, an examination of the metabolic conversion of PCG to EA in solution with ADAMTS-5 revealed that the enzyme does not play a significant role in accelerating the hydrolysis of PCG. These findings suggest that PCG and EA warrant further investigation as targeted therapies for OA given the diverse mechanisms by which they may be able to alter the progression of this disease.

## Figures and Tables

**Table 1:** Characterization of potential ADAMTS-5 binding pockets.

Binding Pocket	P1	P2	P3
	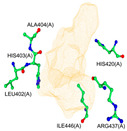	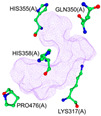	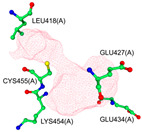
Volume (Å)	728.9	468.1	212.3
Surface (Å)	815.7	1015.1	428
Drug Binding Score	0.77	0.72	0.53
H Donors	10	14	15
H Acceptors	32	40	38
Hydrophobicity	0.60	0.41	0.16
Polar AA Ratio	0.22	0.19	0.43

**Table 2:** Ligand biochemical characterization (* data from the literature) [40].

Ligand	Volume (Å)	XLogP3	PSA (Å)
EA	221.78	1.1	141.33
GA	466.7	1.28	337.3
PCG	800.91	0.17	518.75
HHDP	257.56	0.47	195.97
A2N	419.64	2.61 *	117.12

**Table 3:** Optimal ligand conformation binding interactions.

Ligand	3D Interaction	2D Interaction	Pocket Fit
EA	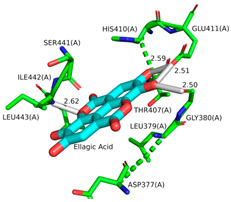	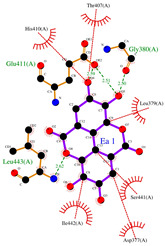	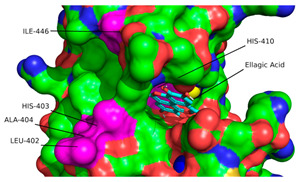
PCG	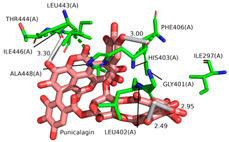	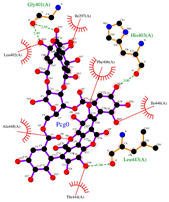	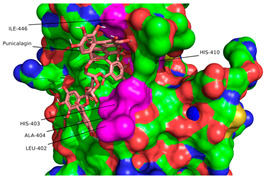
GA	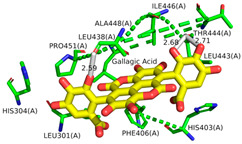	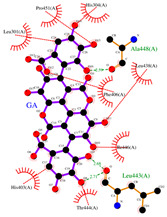	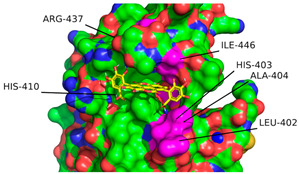
HHDP	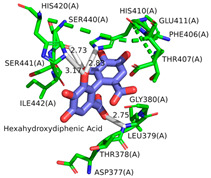	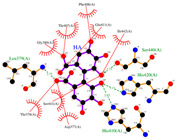	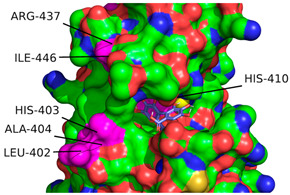
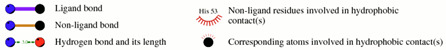			

**Table 4:** Punicalagin hydrolysis quantification of samples incubated with (b) and without (a) ADAMTS-5. Concentration of EA produced quantified as area under the curve of chromatographic peak.

Sample	Area	Ratio (b/a)
a-1	52,658	1.00
a-3	53,495	1.00
a-6	60,182	1.00
a-24	113,690	1.00
b-1	96,634	1.84
b-3	79,002	1.48
b-6	72,104	1.20
b-24	95,802	0.84

**Table 5:** Inputs for grid defining docking region of all ligands.

Grid Box Inputs	
x-points	60
y-points	60
z-points	60
Spacing (Å)	0.375
x-center (Å)	8.063
y-center (Å)	2.49
z-center (Å)	25.01

## Data Availability

The raw data supporting the conclusions of this article will be made available by the authors upon request.

## Abbreviations

The following abbreviations are used in this manuscript:

OA	Osteoarthritis
PCG	Punicalagin
ECM	Extracellular matrix
EA	Ellagic acid
DMOAD	Disease modifying osteoarthritis drug
sGAG	Sulfated glycosaminoglycan
IA	Intra-articular
MAPK	Mitogen-activated protein kinase
ADAMTS	A disintegrin-like and metalloprotease domain with thrombospondin type 1 repeats
HHDP	Hexahydroxydiphenic
GA	Gallagic acid
PSA	Polar surface area
A2N	GLPG1972/S201086
ZBG	Zinc-binding group
DMSO	Dimethyl sulfoxide
RMSD	Root means square difference
PDB	Protein Data Bank
LC-MS	Liquid chromatography–mass spectrometry
SRM	Selected reaction monitoring
